# Comparison between the analgesic effectiveness and patients’ preference for virtual reality vs. topical anesthesia gel during the administration of local anesthesia in adult dental patients: a randomized clinical study

**DOI:** 10.1038/s41598-021-03093-2

**Published:** 2021-12-08

**Authors:** May Almugait, Ammar AbuMostafa

**Affiliations:** Riyadh Elm University, Department of Restorative Dentistry, Riyadh, Saudi Arabia

**Keywords:** Health care, Dentistry

## Abstract

This study aimed to compare the analgesic effectiveness of virtual reality vs. topical anesthesia gel during the administration of local anesthesia (injections to numb the gums) in adult dental patients; as well as to determine which approach is preferred by the patients. Twenty-one adult patients received dental anesthetic injections bilaterally for their maxillary premolars area. We predicted that VR would be more effective than a topical anesthetic gel at reducing pain during injections into the gums. Using a within subject design, each patient received two injections during a single dental visit. Pain was measured after each injection. One side was of the mouth was injected under the influence of the topical anesthesia (TA) 20% benzocaine. The other side of the mouth was injected when the patient was in virtual reality (VR) watching an animated movie using an Oculus Quest® helmet to distract them during the other injection, treatment order randomized. Immediately after each injection, the patients were directed to rate their pain experience using the Wong-Baker Faces Pain-rating Scale (W-BFPS), and to choose which delivery system they preferred. Heart rates were recorded prior to and after the injections using a finger pulse oximeter. Participants reported the predicted pattern of a lower W-PFPS score (less pain intensity) during needle injection while in VR than the injection with topical anesthesia gel, however, the difference was not statistically significant. A statistically significant majority of the participants (p = 0.021) preferred VR to TA. No statistically significant difference heart rate during VR vs. TA was found. Although dental patients reported less pain during VR distraction vs. topical gel anesthetic, the difference was not significant. A statistically significant majority of patients preferred virtual reality over topical anesthesia during their future injections. However, no significant difference in heart rate was found.

## Introduction

Needle phobia is one of the most common phobic conditions. In one recent study, 67% of young pediatric patients had high anxiety before their vaccination^1^, and over 20% of adults aged 20–40 years old have fear of needles^2^. Managing dental fear is considered an important issue in dental practice^3^. Many people who have needle phobia avoid or postpone treatments owing to their fear and anxiety, mainly from dental anesthesia needle pain^4,5^. Rather than attending the recommended regular checkups, patients with dental fear usually only go to the dentist when they suffer pain (e.g., toothache); this increases the chance that their visit to the dentist will cause pain^6^. Moreover, unpleasant early dental experiences can affect patients’ perception of healthcare, can increase pain and suffering during subsequent medical visits, and can reduce preventative healthcare, in turn affecting lifelong health^7^.

Distraction is proving to be an essential technique for reducing pain during short invasive medical procedures^8–10^. Pain perception has a large psychological component in that the amount of attention directed to the noxious stimuli modulates the perceived pain^11,12^. Recently there is growing evidence that virtual reality (VR) can serve as a powerful distraction tool for pain and anxiety control. VR has unique characteristics that makes it a very effective distractor^13^. VR is able to engage different senses simultaneously and induce a sense of presence in the virtual environments (the illusion of “being there” in the virtual world); thus, efficiently diverting attention from painful stimuli^12,14^. The use of immersive VR to distract dental fears patients was first introduced by Hoffman et al. in 2001^15^.

Although previous studies have tested the efficacy of VR as an adjunctive non-pharmacologic analgesic during dental treatments, to date, no study has evaluated its effect on pain perception as a replacement for topical anesthesia to help reduce pain during injection of a local anesthetic. In this study, this will be evaluated as a step toward virtual dental analgesia.

The aims of the study are to compare the analgesic effectiveness of virtual reality (VR) vs. topical anesthesia (TA) gel during administration of local anesthesia in adult dental patients, and to determine which approach the patients preferred to reduce pain of dental injections.

### Primary hypotheses

Using a within subject design, each patient received two injections during a single dental visit. We predicted that (1) VR would be more effective than a topical anesthetic gel at reducing pain during dental anesthesia injections into the gums, (2) patients would prefer VR compared to gel, and (3) heart rate would be lower during the VR condition than during the topical anesthesia gel condition.

## Materials and method

This study was performed in accordance with the Declaration of Helsinki. Twenty-one adult participants visiting the restorative dentistry department in the dental hospital participated in the study, with an age range from 22 to 59 years. The procedures were explained to the participants in detail and an informed written consent form was signed by each participant. The study was approved by the Ethics Committee in the Institutional Review Board (IRB) no. RC/IRB/2019/321 from the Research Center at Riyadh Elm University. The research is also registered in the website of ClinicalTrials.gov with NCT 04919421, date 09/06/2021. The full trial protocol could be accessed through the same website as well.

This study was conducted according to the design of split-mouth randomized controlled single-blinded trials (analyst remained unaware of which group was which). Using a within subject design, each patient received two injections during a single dental visit. Patients received on injection into the gums of the upper premolars on one side; and then the other injection on the contralateral side of the mouth. Each participant was randomly allocated to receive *topical anesthesia* gel before to help numb the gums before one injection, or to go into *virtual reality* during the other injection (treatment order randomized). All dental procedures were done by one person, a senior dentist experienced in administering dental anesthesia. Since it was a within-subject, split-mouth design, each participant served as his/her own control.

## Sample power calculation

Sample power was calculated using the G-Power sample power calculator (Universtat Kiel, Kiel, Germany). Given the split mouth study design, an effect size of 0.75 (large effect size) was assumed and for a power of 0.95, the total number of participants was determined as 21.

### The inclusion criteria

- Class I of the American Society of Anesthesiologists (ASA) as approved by the ASA House of Delegates on October 15, 2014; aged 18 and above, both genders.
- Participants are in good general health, take no medications, and have no contraindications to the use of local anesthetic.
- The ability to understand oral and written instructions.

### Procedure with topical anesthesia gel (TA)

For each participant, the heart rate was recorded immediately before and after the injection using an FDA approved pulse oximeter (SantaMedical SM-165 Fingertip Pulse Oximeter, China). The injection site was dried and isolated using a cotton roll and a small quantity of the topical anesthesia Iolite 20% benzocaine (Dharma Research, Miami, USA) anesthetic gel was applied using the end of an applicator stick directly at the site of penetration for 15 s and then left for 2 min to ensure effectiveness^12^. All the time periods were calculated using Clock App timer available on an iPhone device. The injection of anesthetic solution was performed according to the standard technique mentioned below. Directly after the injection, the pulse rate was recorded a second time.

### Procedure with virtual reality (VR)

For each participant, the heart rate was recorded immediately before and after the injection using an FDA approved pulse oximeter. The virtual reality stand-alone headset used in the study was the 128 GB Oculus Quest^®^ (Facebook Inc. USA). The device was loaded with the animated short movie ‘Henry’ (Oculus Story Studio, USA) and then properly adjusted around the patient’s head and in front of her/his eyes. The volume level was controlled and interpupillary distance (IPD) was adjusted by each participant after providing brief instructions. The movie was played for some time prior to administration of the injection to enable the participant to get involved with the scene. The next step comprised of guiding the patients to turn their heads and adapt with the dentist’s instructions while watching the movie. The injection of anesthetic solution was performed according to the standard technique mentioned below while the participants continued watching the VR movie. Directly after the injection, the pulse rate was recorded a second time.

### Protocol for injection of local anesthetic solution

The injection was made with 1.8 ml Xylocaine 20 mg/ml (DENSPLY Pharmceutical**,** USA); (adrenaline: 1:100.000), delivered in cartridges using a 27—gauge short needle (0.4 × 25 mm, C-K jet). A sterile non-aspirating syringe was used, as recommended for infiltration anesthesia for maxillary teeth^16^.

The anesthetic solution was administered into the buccal sulcus of the treated tooth following a standard technique^16^. The syringe was held parallel with the long axis of the tooth while the tissue was pulled out. The needle was inserted into the mucobuccal fold above the apex of the tooth at a 45° angle with the buccal cortical plate of the bone and with the gauge facing the bone. A few drops of local anesthetic solution were deposited immediately before the needle entered the tissue. After 2 to 3 s, the needle was advanced apically until the bone was contacted without penetration to the periosteum. The rest of the solution was then administered at a slow rate over approximately 1 min. The needle was then withdrawn gently and slowly.

### Patient ratings

After each procedure was performed (TA or VR), the participants were asked to evaluate the degree of pain (primary outcome) that they experienced using the Wong-Baker Faces Pain-rating Scale (W-BFPS), a tool to measure the intensity of pain comprising a scale from 0 to 10. The patient will select a number based on the intensity of the pain, "0" means no pain, "10" means extremely severe pain. Official permission was issued from the Wong-Baker FACES Foundation.

Additionally, each participant was also asked to state his/her preference of delivery system (primary outcome) for future injections. And heart rate will be measured for the participants before and after each injection (primary outcome), using an FDA approved pulse oximeter (Santa Medical SM-165 Finger Pulse Oximeter).

### Risk of bias

To reduce the risk of bias, the following were considered:

• Both procedures were coded and blinded to the one who did the analysis.

• Both types of dental procedures were performed by one person, a senior dentist experienced in administering dental anesthesia to avoid an inter-operator variability influence.

• Special attention was given to keep the syringe out of their line of sight.

• Regarding communication with the participants, the words (*injection shot*, *pain*, and *hurt*) were not used to avoid increasing stress or fears.

### Statistical analyses

Given the subjective nature of Wong-Baker faces pain-rating scale (W-BFPS), non-parametric tests were used to analyze differences in W-BFPS. The normality of the heart rates observed was calculated and was observed to be within acceptable limits of Kurtosis and Skew. Therefore, parametric tests were used to analyze differences in heart rate.

The Wilcoxon Sign rank test was used to compare the W-BFPS between the two methods. The paired *t test* was used to compare the heart rates of the individuals between the two methods. The Spearman’s correlation was used to check the association between W-BFPS and heart rate for each procedure. All tests were performed at p < 0.05.

## Results

### Analysis of the descriptive statistics

The sample was comprised of 21 participants (10 male, 11 female) aged between 25 to 59 years of age. The mean ages for females and males was 35.4 and 34.5 respectively, with no significant difference (p = 0.847).

The patient’s experience of pain with each group was measured using Wong-Baker Faces Pain-rating Scale (W-BFPS). The Wilcoxon Sign Rank test showed that while 10 of the 21 participants had a higher W-BFPS with TA when compared to VR, 5 of the 21 reported a worse experience with VR when compared to TA. There were six individuals who reported the same experience with both procedures (Table 1). These differences were however not significant (Z = 1.662, sig = 0.096).

**Table 1:** Comparisons and ranking of W-BFPS.

		N	Mean rank	Sum of ranks	Z*	Sig
W-BFPS—VR W-BFPS—TA	Negative ranks	5^a^	6.50	32.50	1.662	0.096*
	Positive ranks	10^b^	8.75	87.50		
	Ties	6^c^				
	Total	21				

*Calculated using the Wilcoxon Sign Rank test. Differences are not statistically significant.

^a^W-BFPS—TA < W-BFPS—VR.

^b^W-BFPS—TA > W-BFPS—VR.

^c^W-BFPS—TA = W-BFPS—VR.

**Figure 1:**
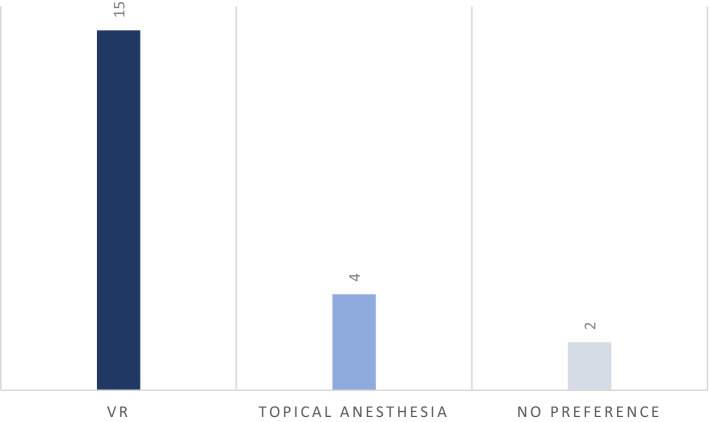
Stated preference of procedure by the population.

When patients were asked which method they preferred, a significant majority (Chi square = 14.124, p = 0.021) reported that they preferred the VR to Topical Anesthesia (Fig. 1).

In order to measure the impact of each injection on heart rate, the heart rates before and after the injection were compared. It was observed that for both procedures the mean heart rate after the procedure was slightly lower than mean heart rate before the procedure (Table 2). However, the paired t test showed that the difference between the heart rate before and after treatment were not significantly different (Table 3).

**Table 2:** Heart rate before and after the treatment for each type of procedure.

		Mean	N	Std. deviation
With VR	Heart rate before	77.7143	21	9.64957
	Heart rate after	75.9048	21	8.53173
With TA	Heart rate before	80.4286	21	12.35950
	Heart rate after	79.0000	21	10.02497

**Table 3:** Significance of difference in heart rate before and after giving the injection for each type of procedure.

		Paired differences					t*	Sig (2-tailed)
		Mean	Std. deviation	Std. error mean	95% confidence interval of the difference			
					Lower	Upper		
With VR	Heart rate before—heart rate after	1.80952	4.02019	0.87728	−0.02044	3.63949	2.063	0.052*
With TA	Heart rate before—heart rate after	1.42857	6.20138	1.35325	−1.39426	4.25141	1.056	0.304*

*Calculated using the paired t test. Differences are not statistically significant.

Overall, females had significantly higher heart rate compared to males at three of the four timepoints that heartrate was measured (Table 4).

**Table 4:** Differences in heart rate according to gender for each procedure.

	Gender	Mean	Std. deviation	t*	Sig
Heart rate before (VR)	Male	73.4000	6.09554	−2.117	0.048**
	Female	81.6364	10.82841		
Heart rate after (VR)	Male	73.9000	6.62403	−1.102	0.311
	Female	77.7273	9.92059		
Heart rate before (TA)	Male	74.9000	9.65459	−2.118	0.048**
	Female	85.4545	12.77782		
Heart rate after (TA)	Male	74.1000	7.29459	−2.369	0.029**
	Female	83.4545	10.35725		

**Differences are significant at p < 0.05.

## Discussion

The present assessor blinded within-subject split-mouth study aimed to evaluate the effectiveness of VR tools in reducing or eliminating the perceived pain during needle injection, compared with topical anesthetic gel to help numb the gums before the injection. Participants reported the predicted pattern of a lower pain ratings (less pain intensity) with VR than with topical anesthesia gel, but the difference between VR and TA was not statistically significant. A significant majority of the participants preferred VR to TA. But contrary to predictions, there was no statistically significant difference heart rate during VR vs. TA.

In the last two decades, there has been increasing interest in the application of virtual reality during dental treatments. The current research advances a step forward from using virtual reality as an analgesic adjunctive tool to using it as an anesthetic adjunctive tool. The study provides some insights for future implementation as it is the first of its kind that tested the effectiveness of virtual reality distraction as a psychological tool to control pain while receiving dental local anesthesia.

Distracting the patient from an unpleasant stimulus may result in decreased pain perception^17^. Studies comparing the ability of interactive and non-interactive VR systems in pain management, found that interactive distraction was more effective^12,18^. This observation supports the view that engaging attentional resources in tasks can interfere with pain processing^19^. Audio, visual, and kinesthetic sensory modalities are all combined in VR, and based on how immersive the stimuli are, a person’s attention will be more or less drained^12,20,21^. In general, manipulations designed to increase the patients illusion of “being there’ in the virtual world, typically also increased how much VR reduces pain.

The Oculus Quest^®^ has several advanced features, of which rotational motion tracking is one of them. This feature is, however, a disadvantage when being used in the dental operatory, if a game is being played while the patient is being treated, as they will not be able to sit still as the virtual reality might impede the positioning of the head, rotation, visual field, and change of direction. Therefore, an animated movie was chosen to be played, instead of games, to ensure patient stillness, and also to overcome any language barrier among the multi-national participants. Atzori et al.^22^ used a custom VR software that allowed participants to use their mouse to interact with the virtual world (mouse tracking), without moving their heads (no head tracking). Although VR movies worked well in the current study, interactive immersive VR is recommended for more painful dental procedures.

The patient’s pain experience with each group was measured using Wong-Baker faces pain-rating scale (W-BFPS). Although the predicted pattern was observed, the differences were however not statistically significant.

When patients were asked which method they preferred, a significant majority (71.4%) favored VR over TA and is indicative of the positive experience patients had with VR. This finding was concurrent with the results of other experimental studies where three-quarters of the patients wished to use video glasses when subjected to future cold pressor tests^23,24^, and 74% of patients reported to prefer wearing video glasses if they were to receive another dental filling^25^.

In the present study, the difference between the heart rate before and after treatment was not statistically significant.

A study which examined the efficacy of VR distraction on pain control during periodontal scaling and root planning procedures (SRP) showed that it was an effective tool^26^. In that previous study, approximately two-thirds of the study population reported that they preferred the VR distraction method during SRP procedures, one-third preferred to watch the movie and only one patient preferred no distraction at all. Not only did patients prefer interacting with the VR environment and report lower levels of pain, but also had significantly lower Pulse Rate and Blood Pressure measurements during the VR experience.

A meta-analysis study^27^ reported that the use of virtual reality glasses is an effective tool for improving behavior and reducing pain perception during the dental treatment of children. Children who used VR eye-glasses behaved better during removal of caries and showed lower pain perception during restoration. Another recent systemic review and meta-analysis^28^, about use of virtual reality for the management of anxiety and pain in dental treatments concluded that VR is a useful tool to reduce pain in children undergoing dental treatment, while there was no significant effect on dental anxiety.

Limitations. This study had a number of limitations to take into consideration. The sample size of the participants was relatively small, further studies with a larger sample size are needed. Additionally, the size of the headset was a bit big and not convenient to perform dental treatment. Smaller headsets have recently become commercially available. Although fear is an important mediating variable, fear was not measured in the current study. Similarly, future studies should measure how much fun patients have during the procedure^10^.

## Conclusion

Despite these limitations, the current study is the first to measure the use of VR distraction to reduce patients discomfort during needle injections. Through this study we can conclude that the effectiveness of virtual reality in reducing pain caused by dental injections is comparable with that of topical anesthesia. Additionally, a significant majority of patients preferred virtual reality over topical anesthesia during their future injections. Previous studies have shown that VR makes painful stimuli more fun^22^, which could make patients more willing to visit their dentist more often. The current results add to growing evidence for the effectiveness of immersive virtual reality as an adjunctive pain control technique. Additional research and development is recommended.
