# Author Correction: Comparison between the analgesic effectiveness and patients’ preference for virtual reality vs. topical anesthesia gel during the administration of local anesthesia in adult dental patients: a randomized clinical study

**DOI:** 10.1038/s41598-021-04480-5

**Published:** 2022-01-11

**Authors:** May Almugait, Ammar AbuMostafa

**Affiliations:** Department of Restorative Dentistry, Riyadh Elm University, Riyadh, Saudi Arabia

Correction to: *Scientific Reports* 10.1038/s41598-021-03093-2, published online 08 December 2021

The original version of this Article contained an error in Affiliation 1, which was incorrectly given as ‘Riyadh Elm University, Riyadh, Saudi Arabia’.

The correct affiliation is listed below:

Riyadh Elm University, Department of Restorative Dentistry, Riyadh, Saudi Arabia.

The original Article has been corrected.

